# Insights Into Learning Among Physical Therapy, Medical, and Nursing Students Following a Simulation-Based, Interprofessional Patient Safety Course

**DOI:** 10.7759/cureus.36859

**Published:** 2023-03-29

**Authors:** Jill S Sanko, Gregory W Hartley, Mary E Mckay, Elsa M Drevyn, David W Mandel, Kathryn S Gerber, Ivette Motola

**Affiliations:** 1 Nursing, Walden University, Minneapolis, USA; 2 Education, MGH Institute of Health Professions, Boston, USA; 3 Department of Physical Therapy, Miller School of Medicine, University of Miami, Coral Gables, USA; 4 School of Nursing and Health Studies, University of Miami, Coral Gables, USA; 5 Gordon Center for Simulation and Innovation in Medical Education, Miller School of Medicine, University of Miami, Miami, USA

**Keywords:** medical students, nursing students, physical therapy students, patient safety, simulation, interprofessional education

## Abstract

As the need for a strong interdisciplinary approach in the delivery of healthcare services becomes increasingly vital, interprofessional education (IPE) is essential to equip healthcare professionals of the future to deliver better care. IPE encounters using simulation-based education can be a powerful tool in inculcating pre-professional students with foundational tools for successful interprofessional work. This qualitative study explores the learning that occurs during IPE encounters that include nursing, physical therapy, and medical students. The results of this work highlight how important IPE encounters are in uncovering and changing cross-disciplinary notions about knowledge, skills, role, and team contributions. Specifically, our analysis demonstrated that there are common misunderstandings about contemporary roles, knowledge, and skills of physical therapists among both nursing and medical students that can be corrected in IPE encounters. Results also demonstrated that careful planning can bolster opinions of the value of activities embedded as part of a larger course. Moreover, planning and attention to the specific educational needs of all students may prevent any group feeling that their educational needs were not fully met across all disciplines. The findings support the consideration that IPE can be an important method to instill foundational interprofessional knowledge, skills, and attitudes to promote a foundation from which to establish strong career-long interprofessional collaborations. It is important to lay foundational interprofessional skills and appreciation of the ‘other’ in pre-licensure curricula, but these efforts should not be limited to only pre-licensure programs and need also to be included as part of on-going professional development education, especially as healthcare education, roles, and responsibilities evolve.

## Introduction

This study is an extension of prior work examining what medical and nursing students learn from, with, and about one another which focused on learning perceptions among and between nursing and medical students [1]. This study expands the scope to include physical therapy (PT) students and more narrowly examines learning perceptions between and among nursing and PT students and medical and PT students and aimed to improve the understanding of the learning that occurs when PT is included in interprofessional education (IPE) with these disciplines In this study, we sought to answer the following: What are the perceptions of nursing and medical students of physical therapists’ roles as members of the healthcare team, the PTs’ scope of practice in the delivery of care, and general knowledge that underpins their care of patients (PT students’ perceptions of the role, scope, and knowledge of nursing and medical students).

Review of the literature

Interprofessional education (IPE) is supported in the literature as a method to hone and develop interdisciplinary practice habits that can lead to reductions in healthcare expenditures, length of stays, and medical errors [2]. Existing evidence demonstrates favorable perceptions of IPE among nursing, medicine, pharmacy, occupational therapy, and physical therapy students and faculty [3]. Systematic reviews on IPE further indicate its ability to foster positive interactions between individuals from varied disciplines [4,5].

The education of healthcare professionals in nearly all disciplines has evolved. For example, all physical therapists (PTs) educated in the United States (US) now receive a clinical doctorate as the entry-level degree (doctor of physical therapy, DPT), and PTs have some form of direct access (the ability to treat without referral) in all US jurisdictions [6,7]. These changing educational requirements and subsequent changes in scope of practice could create misconceptions and confusion within interprofessional teams if unknown. This unawareness has the potential to result in misappropriation of resources, a lack of team efficiency, and suboptimal patient outcomes.

There is a dearth of literature on the perception of medical (MD) and nursing (RN) students, physicians, and nurses on the current level of education, current scope of practice, and expanded roles of PTs in acute care, emergency departments, and primary care. Much of the existing evidence on the inclusion of PT students with MD and RN students during IPE is quite dated and therefore is not reflective of current practice [8-11].

Interprofessional education among student groups is reported to promote long-term retention of appreciation for, and an understanding of each other’s professions. Sytsma et al. reported IPE activities inclusive of PT and MD students in early courses can result in lasting positive attitudes towards IPE and collaborative learning, as well as appreciation for learning with other healthcare professionals and a greater understanding of their roles [12]. Wren et al. conducted an IPE activity during which PT students educated medical students in safe patient handling. In this study, the PT students designed the learning experience and had to learn about the roles and work environment of the MD students. The MD students in turn learned about the expertise and roles of the PT students as it related to safety and safe patient handling [13]. Koplow et al. reported that IPE experiences inclusive of RN and PT students showed increased knowledge about each other’s scope of practice and the recognition of the value of teamwork [14]. Finally, a recent study by Sytsma et al. was designed to assess readiness for interprofessional learning amongst second-year PT and fourth-year MD students before and after a four-hour IPE workshop [15]. Fifty-six percent of PT students reported valuing learning about interprofessional roles and responsibilities compared to 31% for MD students. The authors concluded that IPE success in this case was impacted not only by interprofessional interaction but by content of the IPE activity and timing during the educational process.

While several studies have suggested that IPE, and consequently interprofessional practice, impacts length of stay, patient safety, and total cost of care, few of these studies have included PT students, especially in the context of primary, acute, or emergency care [2,16-18].

Despite findings that are supportive of IPE overall, other results demonstrate variable opinions, perceptions, and attitudes towards other professionals following IPE [3,4]. These data may be in part due to the wide diversity in methods by which IPE courses are administered and evaluated. This represents a gap in what is known about how and under what conditions IPE is most effective for this educational outcome. To this end, this study sought to contribute to what is known about the function of IPE specifically when employing it in the education of nursing, medical, and physical therapy students together with focused attention toward discovering what is learned about, from, and with each other through their respective disciplinary lenses.

## Materials and methods

A multi-methods approach was selected for use due to its ability to yield a more holistic understanding of a phenomenon [19]. This approach rests on the logic of triangulation rather than causal inference thus allowing for a multi-dimensional view of the data [19]. Using more than one method of data collection is an established approach in the social sciences and many formative evaluations of learning to understand and explore why an educational program functions as it does [19]. Given the goal of seeking to improve the understanding of the learning that occurs from, with, and about one another during IPE when nursing, medicine and PT are participants, this strategy was deemed most appropriate. We sought to understand and share insights from an IPE course where Nursing, Medical, and Physical Therapy students participate utilizing two data sources (quantitative and qualitative). This approach allowed for a multi-dimensional view of knowledge acquisition and attitude changes. Few studies are found in the literature exploring IPE learning in these three groups using a multi-dimensional view. Following Institutional Review Board approval from the University of Miami (HRSO 20120053), a retrospective analysis of the post-course evaluations was undertaken.

Subjects

A convenience sample of pre-licensure nursing (n=85; accelerated, second semester of three, bachelor of nursing), doctor of physical therapy (n=59; first semester, terminal year), and medical students (n=140; first semester, third year of four) was used.

Course description

The four and one-half day course was conducted at a private research university in the southeastern United States with nursing, medical and PT schools. Faculty from each of the three disciplines were involved in the writing and review of the scenarios and activities used as part of the course. Course objectives and teaching modalities aligned with, and were guided by the American Association of Baccalaureate Nursing Essentials [20], Quality and Safety Education for Nurses Competencies [21], Interprofessional Educational Core Competencies [22], Accreditation Council for Graduate Medical Education [23], and The Commission on Accreditation in Physical Therapy Education Standards [24]. The course consisted of didactic and simulation-based educational activities that presented concepts of patient safety including teamwork, calling for help, situational awareness, and communication. Activities were completed primarily in mixed discipline teams of five to six students. Establishing the relevancy and validity of the scenarios for all professions is fundamental to learning opportunities. Since the framework of an existing course designed for medicine and nursing was used as the basis for the educational opportunity, all simulation scenarios needed review and revision in order to be applicable to and meet the learning needs of all three disciplines. Non-clinical activities (those that did not require specific clinical knowledge) were also reviewed, but not changed since they were deemed appropriate for all learners due to their lack of discipline-specific knowledge to be able to learn from them (e.g. the puzzle game, Friday Night At the ER, Fine Art of Healthcare workshop). All scenarios used were revised to ensure appropriate learning opportunities for all students.

Unique features of the course included two non-clinical activities, the first being The Fine Art of Healthcare (FAoHC) workshop developed and run by the university’s art museum faculty which teaches visual thinking strategies (VTS). VTS uses art as a platform to develop critical thinking, communication, and observation skills [25]. Embedded in the FAoHC is an exercise where following example setting by the art museum faculty, participants take turns as a lead facilitator using methods taught. The approach utilizes reflection, open-ended questions, and summarization skills to prompt discussions. Following the activity, art museum faculty facilitate a discussion to relate the learned VTS to clinical observation and communication with the patient and other team members. The course also included Friday Night at the ER®, a commercially available team-learning, table-top simulation designed to teach systems thinking and data-driven decision-making. 

The clinical simulation-based activities consisted of several immersive team-based simulations (single and multiple patient scenarios) using simulated patients and/or manikins depicting medical conditions commonly encountered including shoulder pain, hip fracture, and traumatic brain injury. All simulation scenarios began with a pre-briefing and followed with debriefing, facilitated by interprofessional faculty teams. The debriefing approach used a combination of advocacy/inquiry and plus delta. Following the scenario run on the last full day of the course students were provided an opportunity to put their new skills to use and were tasked with debriefing themselves. The two-part, self-debriefed scenario presented intentional overdose with post-fall trauma in an adolescent. Part two followed the patient through to discharge planning where students worked together to develop a comprehensive interprofessional discharge plan appropriate for the patient’s complex medical, physical and social needs. To assist with the self-debriefing, students had the following: a debriefing guide which included a plus delta form and assignment to read the article by Rudolf et al. (2006) 'Debriefing with good judgment' [26], faculty modeled debriefing throughout the week, and VTS which is focused on communication.

Course activities built upon the knowledge and skills presented from prior days using a scaffolding framework, thus allowing learners the opportunity to apply and build onto new knowledge and skills from day to day. The course culminated with “Simulation Olympics™” (Sim-Olympics); an onstage, team-learning ‘competition’ used to emphasize and demonstrate teamwork and communication skills [27]. Two teams were selected by faculty based on team performance in a multi-patient simulation mid-week that was scored using the University of Miami Crisis Resource Management tool (UM-CRM) [27]. The UM-CRM tool had been used by this team for many years and had been psychometrically evaluated as part of its use in prior work [27]. The decision to use this tool is based on its simplicity for use coupled with data to support its ability to capture the construct of teamwork reliably [27]. Students who are not on one of the selected teams are instructed to engage in Sim-Olympics as audience observers and asked to critically evaluate team dynamics guided by, and using the UM-CRM tool in identical scenarios navigated by both teams. Engaging students in the opportunity to evaluate or demonstrate teamwork interactions provided yet another opportunity for students to put their newly acquired skills into action.

Data collection

Post-course evaluations were the data sources for the study. The voluntary anonymous online survey included a series of Likert Scale scored questions probing overall opinions of the course (quantitative) and three open-ended questions (qualitative) asking participants, stratified by their discipline, to describe what they learned from, with, and about participants from the other two disciplines. 

Data analysis - quantitative

Quantitative data was analyzed using descriptive data analysis. Data from the survey were exported from a secure Qualtrics® database (Qualtrics, Provo, UT, USA; 2020) to SPSS Version 27.0 (IBM Corp., Armonk, NY, USA) for analysis. Descriptive statistics were then used to describe perceptions of the course using percentage of agreement/disagreement.

Data analysis - qualitative

**Figure 1:**
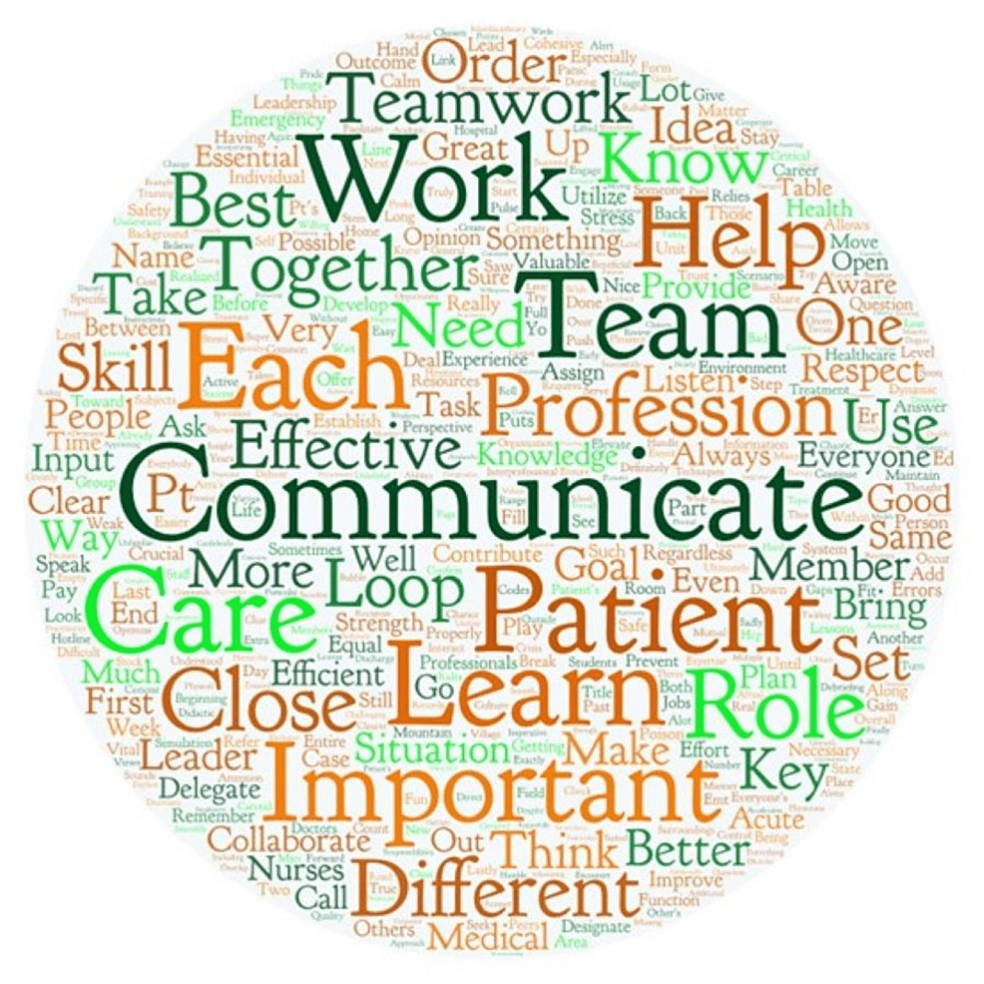
Word cloud depicting what learners learned with each other. Larger words represent greater number of responses.

Following export from Qualtrics, qualitative thematic analysis was utilized to identify and report patterns/themes in the data [28]. Qualitative analysis seeks to achieve an understanding of a specific phenomenon from the perspective of those experiencing it, making thematic analysis an appropriate methodology to explore experiences of multi-professional student groups participating in IPE [29]. Data from participants’ responses to open-ended survey questions were grouped such that it was possible to look at the responses related to what nursing and PT students (and vice versa) learned from, with, and about each other; and what medical and PT students (and vice versa) learned from, with, and about each other. Data were initially read independently by the two researchers to become familiar with the participants’ comments. The coders consisted of a seasoned published researcher with experience in qualitative and mixed/multi-methods research and a PhD student with experience in qualitative research. Following the initial read, the researchers coded data to describe the content. Next, a secondary immersion using an inductive approach was conducted and themes emerged. Additional review of the themes by three additional researchers with qualitative research experience allowed for identification of sub-themes that led to increased understanding of the data from the multiple participant groups’ perspectives. A word cloud (Figure 1) provides a visual representation of the results focused on what was learned with one another. Word clouds are a visual way to represent and analyze textual data based on a simple plot. In word clouds the size of the word represents the number of times that word is used, with the larger the word corresponding to increased frequency [30]. In this case it represented the number of times a given concept was used in answering the question about what was learned with the other disciplines.

## Results

Quantitative findings

Descriptive analysis of Likert scale questions demonstrated consistent opinions regardless of discipline. Collectively, feelings that the course was worthwhile and provided opportunities to gain knowledge and skills were noted universally. Following a similar pattern, analysis showed that most students, regardless of discipline, agreed or strongly agreed the course improved communication, teamwork, and leadership skills. Moreover, an overwhelming majority agreed or strongly agreed that it was helpful to do team-based exercises with different health professions students. Table 1 shows findings for each question by discipline.

**Table 1:** Results of questions from end of course survey Total responses by participant type: Medicine n=112, Nursing n=84, PT n=42. PT=Physical Therapy, SA=Strongly Agree, A=Agree, U=Undecided, D=Disagree, SD=Strongly Disagree

Question	Discipline	SA	A	U	D	SD
The course provided you ample opportunities to gain knowledge and skills.	Medicine	44.64% (n=50)	41.96 % (n= 47)	9.82% (n=11)	3.57% (n= 4)	0% (n=0)
	Nursing	51.19% (n=43)	36.9% (n=31)	5.95% (n= 5)	4.76% (n=4)	1.19% (n=1)
	PT	26.19% (n=11)	52.38% (n=22)	9.52% (n= 4)	9.52% (n= 4)	2.38%(n=1)
Overall, the activities during the course were valuable for your professional development.	Medicine	44.64% (n=50)	40.18% (n=45)	8.93% (n=10)	5.36% (n=6)	< 1% (n=1)
	Nursing	48.81% (n=41)	46.43% (n=39)	3.57% (n=3)	1.19% (n=1)	0% (n=0)
	PT	19.05% (n=8)	47.62% (n=20)	9.52% (n=4)	21.43% (n=9)	2.38% (n=1)
Participation in the course improved my communication skills.	Medicine	44.64% (n=50)	44.64% (n=50)	8.04% (n=9)	1.79% (n=2)	< 1% (n=1)
	Nursing	44.05% (n=37)	47.62% (n=40)	7.14% (n=6)	1.19% (n=1)	0% (n=0)
	PT	38.10% (n=16)	50.0% (n=21)	9.52% (n=4)	2.38% (n=1)	0% (n=0)
It was helpful to do team-based exercises with members of a different health profession	Medicine	63.39% (n=71)	33.93% (n=38)	2.18% (n=3)	0%(n=0)	0% (n=0)
	Nursing	55.95% (n=47)	40.48% (n=34)	2.38% (n=2)	1.19% (n=1)	0% (n=0)
	PT	50% (n= 21)	45.24% (n=19)	2.38% (n=1)	2.38% (n=1)	0% (n=0)
Participation in the course improved my teamwork skills	Medicine	46.43% (n=52)	47.32% (n=53)	4.46% (n=5)	<1% (n=1)	< 1% (n=1)
	Nursing	51.19% (n=43)	44.05% (n=37)	2.38% (n=2)	2.38% (n=2)	0% (n=0)
	PT	35.71% (n=15)	52.38% (n=22)	7.14% (n=3)	4.76% (n=2)	0% (n=0)
Participation in the course improved my leadership skills	Medicine	41.07% (n=46)	44.64% (n=50)	10.71% (n=12)	1.79% (n=2)	1.79% (n=2)
	Nursing	38.10% (n=32)	45.2% (n=38)	11.9% (n=10)	4.76% (n=4)	0% (n=0)
	PT	30.95% (n=13)	42.86% (n=18)	23.81% (n=10)	2.38% (n=1)	0% (n=0)

While most students had very positive opinions of the course, there were areas where the percentages of agreement differed substantially (Table 1). One example is found in the data from the question asking about the course activities overall. When asked about course activities 44.64% of the medical and 48.81% of nursing students strongly agreed that the course activities met expectations and were worthwhile, however just 19.05% of PT students strongly agreed that overall course activities were worthwhile. Similarly, this pattern was noted in the data for several other questions (Table 1).

Qualitative findings

Results following thematic analysis of the submitted responses are summarized below.

*Question: What are the lessons you learned about the nursing students? *From PT students: Analysis of this question highlighted several themes, namely a newfound appreciation for the knowledge that nursing students possess specifically in the areas of pharmacology/medications, medication administration, and hands-on patient care. Respondents remarked about the leadership abilities, communication skills, and teamwork competencies evident in the nursing students. PT students were consistently impressed with the amount of clinical experience the nursing students brought to encounters. “*They are able to take charge in emergency situations. They have a vast knowledge of drugs/medications.” *And,* “I was impressed by the clinical skills of the nursing students. In particular, their ability to evaluate patients in emergency settings and their responsiveness in crisis*.”

*Question: What are the lessons you learned about the medical students? *From PT students:Analysis of this question highlighted themes including medical students’ knowledge in the areas of pharmacology, physiology, diagnoses, and pathology. PT respondents remarked about value of the medical students as a member of an interdisciplinary team. The PT students shared the perspective that the medical students lacked clinical experience/education (related to where they were in their matriculation) noting that medical students would likely obtain greater confidence with more clinical exposure. Themes clustered around the notion that due to the limited clinical exposure the medical students had prior to the course, the medical students were hesitant to act in some situations, especially if the patient condition was complex (e.g., had multiple organ systems issues). Exemplar quotes demonstrative of this finding included: *“The medical students have not had clinical experience yet. They appeared unsure of themselves at times and hesitant to take the lead,”* and *“The medical students lack the clinical experience and still have a lot of education to finish up. Their knowledge is there but the confidence is still missing.”*

Despite the noted clinical skill gap, there was recognition of the value that the medical students brought to the encounters. Specifically, their ability to lead and be strong team members, and their willingness to collaborate.

“It was clear that the medical students have a strong knowledge in pharmacology and the physiology of the body. It was nice to work together and learn more about their skill set and how they can help us just as much as we can help them professionally. Many of the medical students I interacted with seemed to be very down to Earth and happy to work together.”

Several respondents commented that the medical students did not appear to have a firm understanding of the role of a physical therapist or their clinical utility in patient care (in the context of course activities/scenarios). One particularly direct illustrative comment was, *“They [medical students] have no idea what a physical therapist does and were very closed off to conversation to learn more about it.” *Finally, there were several comments on medical students’ personalities and personal attributes;* “they are not as cocky and arrogant as we think,”* and *“they are eager to learn and look for help when needed.”*

*Question:* *What are the lessons you learned about the physical therapy students? *From nursing students: Analysis of this question mirror themes found in other question responses. The nursing students noticed that the PT students were knowledgeable, skilled, and an asset to the team; *“I never knew how much knowledge they [PT students] had about identifying diagnoses. They’re very much an asset in the clinical setting.”* The nursing students commended the PT students on their willingness to assist and on their teamwork prowess.

Additionally, the nursing students’ responses drew attention to the distinct skills and knowledge the nursing students noticed the PT students brought to team encounters, including patient positioning techniques, patient movement approaches, and discharge planning knowledge, e.g., *“I learned that PT students aren't just there to help patients walk, but rather they play a major role in how a patient recovers and goes on with their life after a traumatic illness or event.” *

While the bulk of the themes keyed in on positive attributes that were observed, there were some responses that brought awareness to areas where PT students were not well-suited or lacked the requisite skills to fully participate, *“The PT students have a lot to offer, and are a valuable asset but not necessarily in emergent scenarios.”*

From medical students: Findings from this question demonstrated medical students improved understanding of the role of PT and the wider scope of practice that PTs have; *“I learned that PT students do way more than just MSK [musculoskeletal].”* The medical students consistently noted the breadth of knowledge the PT students had which extended beyond solely the musculoskeletal system; *“They [PT students] have an area of expertise that medical students do not. It’s useful,” and “Physical therapy students also have a ton of knowledge and a lot to contribute to patient care/management.”* The medical students further noted the knowledge PT students had regarding rehabilitation, outpatient care, and discharge planning; *“[The PT students were]…very knowledgeable about home safety assessment, thorough in discharge planning [sic].”* The medical students noted that the PT students were very knowledgeable about anatomy and patient mobilization; *“They [PT students] are the experts when it comes to structural and anatomical diagnosis and management.” *Medical students commented that although the knowledge base of the PT students overlaps with theirs some of the PT’s knowledge base is different; *“They [PT students] have tons of knowledge not taught to us [medical students].”*

*Question: What are the lessons you learned from the physical therapy students?* From medical students:The medical students learned from the PT students about discharge planning, patient mobility, and the importance of evaluating a patient’s environment. Respondents remarked about the specific PT knowledge including expertise about fractures, braces, weight-bearing exercises, positioning, non-pharmacological pain management, wound care, and the importance of follow-up care.

“I learned all about the discharge process of a patient with a muscle or fracture-related injury and how complex it can be from mobilizing the joint or muscle to medications and the extent of lifestyle modifications needed once out of inpatient or outpatient [sic] to ensure patient safety and simplify moving around.”

From nursing students: Data from this question demonstrated that PT students provide a well-rounded and different perspective that is important to positive patient outcomes and bolsters effective collaboration.

“Realization that we really don’t know how much the other disciplines know. The assumptions we make end up limiting our own powers and ultimately weakens the entire team. We all must be open and respectful to each other’s knowledge, skills, and perspective.”

Additional themes included recognition that PT students maintain broad general knowledge as well as specific expertise about patient positioning, teamwork/collaboration being vital, and the importance of communication (specifically use of careful listening and closed-loop communication). Capacity-related themes found were an expanded knowledge of the role of physical therapists and the PT’s scope of practice, and the vitalness of PTs as members of interprofessional teams.

“I learned that each profession would be very weak if working alone, but as a whole, there is a better quality of patient care.”

*Question: What are the lessons you learned from the nursing students? *From PT students: PT students commented that nursing students have good hands-on skills, are safety-oriented, particularly when it came to hand hygiene and medication administration and were effective in communication and general mistake prevention. *“They [nursing students] were a constant reminder to practice proper hand hygiene. They also modeled effective communication.” *and* “They can help prevent costly mistakes with patients.”*

Other themes found were the PT students’ avowal that the nursing students were effective in dealing with families’ and patients’ social challenges. *“They [nursing students] helped demonstrate how to deal with a combative/hysterical family member.” PT students also, through their interactions with the nursing students, developed an improved clarity about nurses’ scope of practice, “I didn’t fully understand the scope of practice of nurses, so it was nice spending time with nursing students to understand their role more completely."*

*Question: What are the lessons you learned from the medical students? *From PT students: The PT students noted the leadership qualities of the medical students as well as their knowledge in the areas of pharmacology, pain management/diagnosis, and their ability to work through a differential. *“I learned successful leadership skills and strategies for differential diagnoses in the acute care/emergent setting.” “I learned a lot about the systematic way they [medical students] go through differential diagnosis.” “I think I just learned some different thoughts to have when a patient presents with certain pain/presentation.” *While most of the PT students’ reflections highlighted positive lessons learned, some PT students felt the medical students had a poor understanding of the role of PT in acute care or emergency departments; *“They do not know a lot about the PT aspect of care.” “They really don’t understand where we [PT] fit in for certain patients.”*

*Question: What are the lessons you learned together with the other profession that you will remember as you embark on your career?: *This question illuminated a great deal about learning during IPE encounters (Figure 1). Many of the concepts which emerged using this graphical analysis tool overlapped those that appeared in the data when analyzing the other open-ended questions, including the theme around how students gained an appreciation for both the specific and shared knowledge of each discipline, the importance of communication, and the benefit of understanding the professional roles of other healthcare providers. Beyond these imbricated themes, others emerged including students’ developing a deeper appreciation for the utility of interprofessional teams and the value of teamwork, quality leadership, and collaboration for optimal patient outcomes. Additionally, themes around respect for others, placing trust in your team, and being open to others’ input surfaced. The following quote is one example of this: *“Ultimately, caring for patients requires a team and every member has something different to contribute. It’s important to get input from all team members and to recognize when someone may have more experience than you in a certain area. You also need to be open to other members’ input in order to make effective use of it!”*

## Discussion

Our findings were consistent with prior published work, but also highlight where further efforts in IPE still need to occur. Overall, students responded in a positive manner and appear to learn and grow during these encounters. IPE can serve as a catalyst of quality team interaction leading to improvements in healthcare if introduced early and is led by experienced faculty [5]. Interdisciplinary team approaches during the delivery of patient care are known to reduce systematic errors, healthcare costs, and unwarranted care, therefore establishing early and solid foundations for interprofessional work collaborations is vital to successful patient outcomes [2,16] For this to occur a comprehension of each healthcare professional’s role, full scope of practice, and respect for what each contributes to the clinical team is requisite. Without this critical understanding, healthcare providers may be prevented from working to the full extent of their scope of practice, thus destabilizing healthcare and in turn creating healthcare environments that fail to fully utilize the value of team-based care and collaboration. The common misunderstandings of professional roles, knowledge and skills were findings noted in this study and may shed light on a more widespread lack of interprofessional knowledge around other healthcare professionals’ roles. This study revealed a knowledge gap among medical and nursing students regarding the expertise and skills of PTs while demonstrating the value of IPE as was also the finding of a systematic review by Guraya and Barr [5]. 

Implications of the findings of this study include the need to provide more curricular opportunities for IPE involving PT alongside other health professionals [5]. Practicing PTs may further consider increasing exposure at non-PT conferences, fostering meetings to advocate for PT, and highlighting their important role in patient outcomes and help clarify misunderstandings of their role and knowledge. Strong PT advocates are critical to improving other healthcare professionals’ understanding about the PT profession and its resource utilization to improve patient outcomes. On the flip side, non-PT providers need to be open to learning about how their PT colleagues round out the continuity of care and assist to bolster the overall quality of care for improved patient outcomes.

Analysis indicated that students from all three included disciplines valued the experience provided as well as the multi-professions, team-based activities. Importantly, it appears the opportunity allowed students to gain a greater understanding about each provider’s unique and overlapping roles and expertise. This broadening of cross-disciplinary metacognition of the ‘other’ may provide an important foundation from which to establish early and effective interdisciplinary relationships. Further, as also noted in Guraya and Barr's study, it may also mitigate stereotypic opinions of each others' professional roles and knowledge, thus setting the stage for an ‘equal turf’ mentality from the outset of careers. Irrespective of discipline, all participants reported the IPE activities they engaged in were of value for their professional development. These findings along with other studies' conclusions leads to the notion that interprofessional training ought to begin early in professional schools [5,12].

This study adds to the evidence supporting IPE and its potential to foster cross-disciplinary respect and collaboration. The importance of building these connections among future healthcare professionals who will enter a field where interprofessional practice is pivotal to quality care, mitigation of errors, and cost-containment cannot be understated and is supported by other work in this field [16]. This study supports the premise that IPE provides students with a platform to learn from, about, and with each other, as well as a space to correct inaccurate notions about the ‘other.’ As demonstrated, carefully planned IPE may serve to increase learners’ knowledge of other disciplines and simultaneously teach important skills related to other topics as was done in our course (e.g., patient safety, systems thinking, communication).

Each profession brings unique skills and perspectives to the delivery of care; it is detrimental to the patient when a lack of cohesiveness and communication within the clinical setting exists. IPE assists in preparing students for their professional role as a member of a interdisciplinary team. The ability to know how to effectively collaborate and communicate across professions provides a better opportunity for all to be more efficient and effective in their own roles so that they can deliver high-quality care while maximizing system resource utilization. 

Strengths and limitations

Strengths of this study include collaboration among a multidisciplinary team of experts at a research institution versed in the delivery of simulation-based education in an acute/emergent care IPE context, and the use of validated IPE activities. Data collection from a single educational setting, as well as a single IPE safety course were some of the other limitations. Because all students came from the same organization, the ability to generalize the findings of this study may be limited, however assuming similar curricula in other institutions, broad applicability is possible. Moreover, this study only examined the perceptions and outcomes of an IPE course from the lens of nursing, medicine, and PT; thus, conclusions about how inclusion or exclusion of other typical members of an interprofessional team cannot be made.

Finally, this was the first year where PT participated in an already well-established IPE course that had previously included only medical and nursing students. While interprofessional teams were used to re-design multiple validated simulation scenarios to include PT, post-course analysis of the strengths and weaknesses of the modified scenarios uncovered that further revision was needed to ensure that all activities met the educational needs of all participants. This illustrates that educators must be mindful when designing IPE courses such that all activities provide equal opportunities for all to acquire knowledge and skills based on current practice standards and jurisdictional scope and that the educational design process is iterative. 

## Conclusions

As interprofessional practice collaboration continues to become more critical in healthcare, so does high-quality IPE that includes all potential members of a healthcare team. An appreciation of professional roles, scopes of practice, and contemporary practice standards increases the likelihood that care will be efficient, effective, necessary, and safe; all of which can be learned during well-planned IPE. IPE benefits all aspects of the healthcare system including patients, families, organizational stakeholders, the professionals delivering care, and society as a whole. It will be up to current and future educators to continue to provide opportunities to build and sustain vital, non-technical skills in the healthcare workforce.

While this work provides a rare qualitative view into the nuanced learning that is acquired during IPE encounters, more research is needed to further explore the effectiveness of IPE in different contexts, with different professional groups, and on learning outcomes. Further efforts should be made to explore ways that IPE continues beyond school. Efforts to impart IPE concepts and mindsets will fall short if the opportunities to hone skills are only offered in pre-licensure programs. These proficiencies, like all skills, degrade if continued opportunities to practice them are not made available.
